# Project VITAL: A Statewide Initiative Using Technology to Impact Social Isolation

**DOI:** 10.1093/geroni/igab046.529

**Published:** 2021-12-17

**Authors:** Sam Fazio

**Affiliations:** Alzheimer's Association, Chicago, Illinois, United States

## Abstract

Project VITAL leverages customized technology to enhance connection, engagement, education and support to individuals with dementia and their caregivers. The goal was to positively impact social isolation, stress, and well-being and help mitigate the effects of isolation experienced during the pandemic and beyond. Phase One included three components designed to impact connection, engagement, education and support. It’s Never Too Late provided 300 customized tablets to 150 care communities to facilitate connections to family members and increase individualized, person-centered engagement. Project ECHO, a video-based learning platform, educated and supported care community staff. Virtual education and support were offered to family caregivers at home. Phase Two provided the program to an additional 150 care communities and added online professional training/certification. Phase Three focuses on 150 family caregivers and individuals with dementia living at home. In addition to connection and individualized engagement, family caregivers now had direct access to virtual programs and services.

